# A Report of Rosai–Dorfman Disease in an Adolescent

**DOI:** 10.1155/2022/9571400

**Published:** 2022-05-26

**Authors:** Emmanuella Amoako, Kwadwo Apeadu Danso, Rosemary Sefakor Akuaku, Kofi Ulzen-Appiah

**Affiliations:** ^1^Department of Paediatrics and Child Health, Cape Coast Teaching Hospital, Ghana University of Cape Coast, School of Medical Sciences, Cape Coast, Ghana; ^2^Department of Paediatrics and Child Health, Cape Coast Teaching Hospital, Cape Coast, Ghana; ^3^Department of Pathology, Cape Coast Teaching Hospital, Ghana University of Cape Coast, School of Medical Sciences, Cape Coast, Ghana

## Abstract

Rosai–Dorfman disease (RDD) is a rare disease of unknown cause. It is a benign self-limiting condition characterized by the accumulation of activated histiocytes in the sinusoids of lymph nodes and/or extranodal tissues. Massive cervical lymphadenopathy as the initial manifestation tends to raise the initial odds in favour of a lymphoma, and thus reducing the threshold to performing a simple biopsy cannot be overestimated. Herein, we report a 13-year-old adolescent who presented with a progressive posterior left-sided neck swelling. Our diagnosis of RDD was established by demonstrating emperipolesis in histology and S100 positivity in immunohistochemistry as stated in the literature. Although the condition is known to be self-limiting, evidence from the literature and our case management shows that medical therapy can hasten remission in pediatric cases.

## 1. Introduction

Rosai–Dorfman disease (RDD) is a rare, benign lymphoproliferative disease of non-Langerhans cell histiocytosis. This condition was first described by Pierre-Paul Louis Lucien Destombes in 1965 in four French patients presenting with “adenitis with lipid excess” and was referred to as “sinus histiocytosis with massive lymphadenopathy” [1, 2]. In 1969, Juan Rosai and Ronald Dorfman characterized this condition as a distinct clinicopathological disorder and has since been termed the Rosai–Dorfman disease [3]. RDD has a worldwide prevalence of approximately 1 : 200000 with a slight male predilection (1.4 : 1) and is more common in blacks and Caucasians [4]. The disease can be observed at any age but mainly manifests in children, adolescents, and young adults. It is a rare cause of rapidly progressive lymphadenopathy in children [5].

Patients with RDD typically present with fever, leukocytosis, elevated ESR, night sweats, weight loss, and lymphadenopathy affecting the cervical lymph nodes [3, 6]. Although the disease has a predilection for the lymph nodes in the head and neck, extranodal manifestations are also observed in 43% of cases, with common sites including the skin and soft tissue, the central nervous system (CNS), bone, and the gastrointestinal tract [6–9].

The aetiology of RDD is enigmatic; however, RAF/MEK/ERK pathway mutations have been implicated, suggesting that the disease may be clonal in some cases [10]. Previous studies have suggested that viruses such as HHV, HIV, EBV, and CMV and bacteria including *Brucella* and *Klebsiella* spp. have been linked to the disease [6, 11, 12].

Radiological findings of RDD are non-specific, resembling lymphoma. Diagnosis is based on clinical manifestation and ultimately on histopathological examination [10]. Classically, histiocytes in intranodal Rosai–Dorfman disease engulf small lymphocytes, plasma cells, and erythrocytes without phagocytosis. This process, known as emperipolesis, can be identified on routine haematoxylin and eosin stains. Immunohistochemical staining is reactive for S100, variably CD68 positive, and reliably negative for CD1a and langerin. Pathological features may be associated with non-Hodgkin and Hodgkin lymphoma where the lymphoma and RDD can either precede or follow each other and occur in the same node [11].

Immunologic diseases coexist with RDD in up to 10% of cases. These include systemic lupus erythematosus, idiopathic juvenile arthritis and autoimmune haemolytic anaemia [6], and a case of a rare genetic disorder of the immune system, RAS-associated autoimmune leukoproliferative disease (RALD) [13].

There is no specific treatment for RDD. However, management is largely dependent on the individual clinical manifestations. Spontaneous remission occurs in about 20–50% of cases. Surgery to remove all cancerous tissue can be curative if the disease is unifocal. Immunomodulatory therapy, chemotherapy, radiotherapy, corticosteroids, and sirolimus have all been used with variable success [6].

We present a case of RDD diagnosed and managed medically at the Cape Coast Teaching Hospital in the Central Region of Ghana.

## 2. Case Presentation

A 13-year-old male adolescent presented to the emergency unit in June 2021 with a 3-month history of a progressive posterior left-sided neck swelling with associated pain. According to the Wong–Baker Faces Pain Rating Scale, he chose 10 to represent the severity of the pain. There was no history of fever, chronic cough, night sweats, weight loss, or abdominal distention. At the onset of the illness, he reported to a herbal clinic; however, he did not notice any form of improvement. He was then referred to our facility for expert management. His past medical history was unremarkable. There was no positive family history of similar swelling. On physical examination, he was not pale, afebrile, and weighed 55 kg, and he had a tender left posterior cervical lymph node measuring 10 × 10 cm, firm with a nodular surface, immobile, and attached to the underlying muscle. There was also a left supraclavicular lymph node which measured 8 × 9 cm with similar characteristics. There was no other peripheral lymphadenopathy. His liver span measured 14 cm with tenderness. Other aspects of the systemic examination were unremarkable. Initial differential diagnoses entertained were tuberculous lymphadenitis and lymphoma. FBC showed an elevated WBC of 31.95 × 10^9^/L with an accompanying increase in neutrophils of 27.15 × 10^9^/L, moderate normocytic normochromic anaemia with Hb of 10.8 g/dL, normal platelets, and elevated ESR 110 mm/hr. Serum LDH was elevated at 536, with a normal uric acid level and a negative result for hepatitis B surface antigen, hepatitis C viral, and HIV screening. An ultrasound of the neck swelling showed hypoechoic, ovoid to round solid mass with irregular margin, measured 3.4 × 3.5 × 4.8. INR done was normal. AP and lateral X-rays of the neck and chest, respectively, showed normal findings. The mother could not afford an initial abdominal USG. He received IV ceftriaxone 2 g for 5days. Adequate pain control was achieved with a combination of IV paracetamol 1 g TDS and IV morphine 10 mg QID for 10 days, respectively, in addition to a 4-day-course of tablet ibuprofen 400 mg QID. The surgical team performed the excision biopsy on day 12 of admission. He was discharged 2 days after the procedure with oral formulations of the analgesics and syrup lactulose.

**Figure 1:**
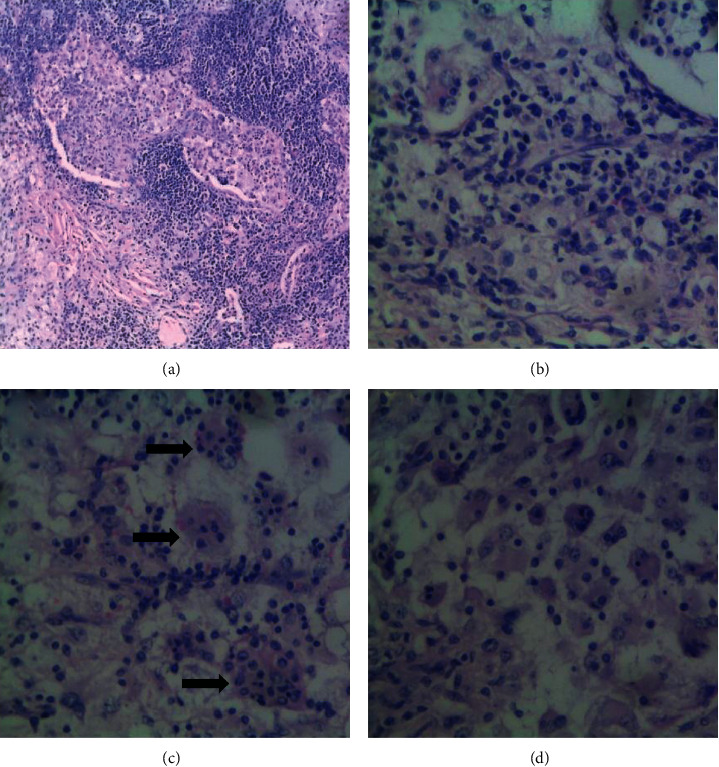
(H&E x 400)- shows effacement of nodal architecture by diffuse sheets of histiocytes with abundant eosinophilic cytoplasm (a) and surrounding lympho-plasmacytic infiltrate (b). Areas where the histiocytes engulf intact chronic inflammatory cells are referred to as emperipolesis (black arrows) (c).

**Figure 2:**
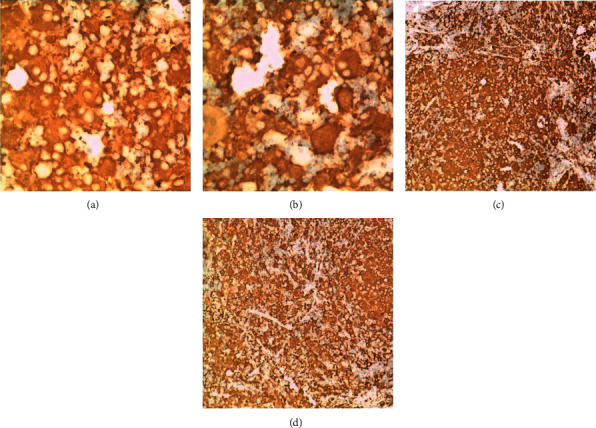
(x100-C&D and x400-A&B) - shows diffuse cytoplasmic staining of histiocytes for S100 (2C&2D) with negative staining for engulfed inflammatory cells- emperipolesis (2A&2B).

Routine haematoxylin and eosin-stained sections of the lymph node biopsy showed effacement of the nodal architecture by diffuse histiocytes with surrounding lympho-plasmacytic infiltrate in areas the histiocytes engulf intact lymphocytes suggestive of emperipolesis (Figure 1). Immunohistochemical staining for S100 was done to rule out Langerhans cell histiocytosis and confirm a diagnosis of Rosai–Dorfman disease (presence of emperipolesis), and the section showed diffuse reactivity of the histiocytes for S100 with negative staining of the lympho-plasmacytic infiltrate (Figure 2).

Due to the lack of funds, the CD1a marker and serum immunoglobulin were not tested for. He showed up for his first review in 3 weeks, where he received a tablet of albendazole 400 mg stat and was commenced on oral prednisolone 1 mg/kg daily in addition to pain medication. On his second review in a week, findings from a scrotal USG were normal; abdominopelvic USG findings revealed an enlarged liver measuring 16.8 cm with normal echotexture, no focal lesions seen, and multiple benign looking mesenteric lymph nodes in the left iliac fossa (largest measuring 0.7 cm). All other organs were sonographically normal. The size of the cervical lymph node had reduced by about 50% (5 cm × 6 cm), and the pain level had reduced to a score of 2. The dosage of prednisolone was reduced to 0.5 kg/mg daily, and he was scheduled for the next review in 5 weeks' time. Unfortunately, the patient has been lost to follow-up despite numerous telephonic requests.

## 3. Discussion

Massive cervical lymphadenopathy is the hallmark of this condition. The axillary, inguinal, and mediastinal nodes may be affected [14]. Fever which is the most commonest associated systemic symptom can be found in just about 30% of cases [15]. It is therefore not surprising that a history of a long-standing fever was absent in this case. The other popularly known systemic symptoms of weight loss and night sweats were also negative. The examination findings of the affected lymph nodes can range from being mobile and discrete to large, multinodular, and adherent to the surrounding structures [16]. Because enlarged unilateral or bilateral lymph nodes are a frequent manifestation, in isolated or multiple regions, lymphoma may be clinically suspected [17]. This dilemma is usually resolved with close attention to the H&E morphology and immunohistochemistry. The monomorphic population of lymphoid cells is specific for lymphomas. Classic Hodgkin's lymphoma will usually show more typical Reed–Sternberg or Hodgkin's cells which are positive for CD30 and CD15 and negative for S100.

Fine needle aspiration cytology is a useful and cost-effective tool in tissue sampling; however, the odds of misdiagnosis are high. A review of the literature of 49 previously published cases of RDD with FNA cytology by Shi et al. [18] showed that even more misdiagnosis of RDD by FNA cytology occurs in extranodal than in nodal disease. Biopsy confirmation revealed that 3 out of 25 (12%) lymph node aspirations were misdiagnosed or inconclusive and 6 out of 12 (50%) extranodal aspirations were misdiagnosed. The learning points here could be that FNA is not infallible and performing an excisional biopsy is advantageous to obtain adequate tissue for histopathological analyses to achieve higher diagnostic accuracy. Concerning our case, tissue sampling was obtained by excision biopsy, rendering the diagnostic accuracy high.

The phenomenon of emperipolesis seen on cytological examination is the hallmark of RDD. It implies the engulfment by histiocytes of lymphocytes, monocytes, and even erythrocytes, without subsequent destruction of these cells. Immunohistochemistry is also employed to help exclude other diseases. RDD shows immunoreactivity of histiocytes for S100 and CD68. This patient's diagnosis was confirmed based on the histological features and S100 positivity. Perhaps the most important differential diagnosis of RDD is that of Langerhans cell histiocytosis (LCH) as both are S100 positive. In RDD, the CD1a stain is negative [10]. Also, the abundant eosinophils in LCH make it morphologically different from RDD [19]. The absence of this feature, in this case, solidifies the diagnosis of RDD. The lack of a CD1a test in this case, however, is a pitfall. Other differential diagnoses can include infectious lymphadenitis and reactive lymphoid hyperplasia with sinus histiocytosis, where emperipolesis is absent and S100-protein stain is negative.

An elevated ESR and polyclonal hypergammaglobulinemia have been reported in up to 90% of cases [20]. The former was observed in our case; however, the latter investigation was not performed in this case. This was another pitfall in our investigative workup.

Due to the self-limiting nature as well as the rarity, no standard treatment protocol has been made for RDD. As a general principle, treatment is best tailored to the individual clinical circumstances. Treatment options include expectant monitoring, steroids, chemotherapy, and surgical debulking [6]. Francesco di Dio and colleagues [21] analyzed 32 case reports of pediatric RDD published in medical literature, focusing on medical therapy. No treatment was used in 9 cases; the outcomes were complete regression in 2, clinical improvement in 3, and clinical stability in 5 patients. For those placed on steroids, complete regression, clinical improvement, and clinical stability were observed in 3, 4, and 0 patients, respectively. Similarly, out of 11 patients placed on chemotherapy, 4, 5, and 2 of them had complete regression, clinical improvement, and clinical stability, respectively. From these data, we could agree with the opinion he gave in his write-up that even though a good percentage (20–50%) of RDD cases are self-limiting, medical therapy might hasten remission. One viable approach is to treat to the best-observed response, followed by a slow taper [6]. This was a strategy we employed in our case as we observed a 50% reduction in the size of the cervical lymph node after 3 weeks of steroid therapy. The basis of administering albendazole before initiating the steroids in our case is the endemic nature of strongyloidiasis in our geographical location. It, therefore, makes it necessary to deworm our patients before subjecting them to long-term immunosuppressive therapy [22]. Unfortunately, further assessment could not be done as he was lost to follow-up. Chemotherapy was shown to be effective as well in Francesco's study; however, clinical response to the same protocols did not appear to be constant. Surgery has also shown very promising results, and complete resection has been found to be curative as Pulsoni et al. showed that 8 out of 9 patients obtained complete remission [12]. One point of note, however, is the possibility of a recurrence [23].

Regular long-term follow-up is indicated, which should include assessment of the progression of the disease, screening for concurrent diseases such as lymphomas [11], and assessing the response to treatment. The lack of long-term follow-up data on our patient was also a limitation of this case. Reassurance and counselling to ameliorate the stigma and aesthetic aspects should also be a priority of the treating physician, along with an explanation of the good prognosis of the condition.

## 4. Conclusion

The knowledge of the presentation of RDD with an understanding of the differential diagnosis is important in its clinical management. Medical therapy has been found from literature and our case management to hasten remission in pediatric cases.

## Data Availability

The data used to support the findings of this study are included within the article.
